# Trichofolliculoma of the external auditory canal: a case report and review of the literature

**DOI:** 10.1093/jscr/rjac147

**Published:** 2022-04-12

**Authors:** Matthew I Saleem, Tristan Tham, Brent Ponder, Alexandros Georgolios

**Affiliations:** Department of Otolaryngology, Donald and Barbara Zucker School of Medicine at Hofstra/Northwell, New York, NY, USA; Department of Otolaryngology, Donald and Barbara Zucker School of Medicine at Hofstra/Northwell, New York, NY, USA; Poplar Bluff Regional Medical Center, Poplar Bluff, MO, USA; Poplar Bluff Regional Medical Center, Poplar Bluff, MO, USA

## Abstract

Trichofolliculoma is a rare tumor that arises from immature hair follicular tissue. In this report, we present the case of a 51-year-old man with a trichofolliculoma in the left external auditory canal. Uniquely, there was no prior trauma in this patient, despite previous trauma being hypothesized in the etiology of this tumor. The lesion was excised using microsurgical instruments and the tumor was closely adherent to the cartilage of the external auditory canal. Histopathology following excisional biopsy confirmed the diagnosis.

## INTRODUCTION

Trichofolliculoma (TF) is a rare pilar tumor which lacks maturation of the hair follicle and represents an intermediate stage of differentiation between simple hyperplasia of the hair follicle (hair follicle nevus) and trichoepithelioma [1]. In adult patients, the lesion usually presents in the face, occasionally in the scalp or neck, but it is also reported in the auricle, genital area, lips and vulva [2].

Auricular involvement is extremely rare, and to the best of our knowledge only three cases of ear canal TFs have been reported in the English literature [3–5]. Histopathological examination is required to confirm the diagnosis. Complete excision of the tumor is the treatment of choice. We present the rare case of a TF located on the external auditory canal in a 51-year-old male patient.

## CASE REPORT

**Figure 1:**
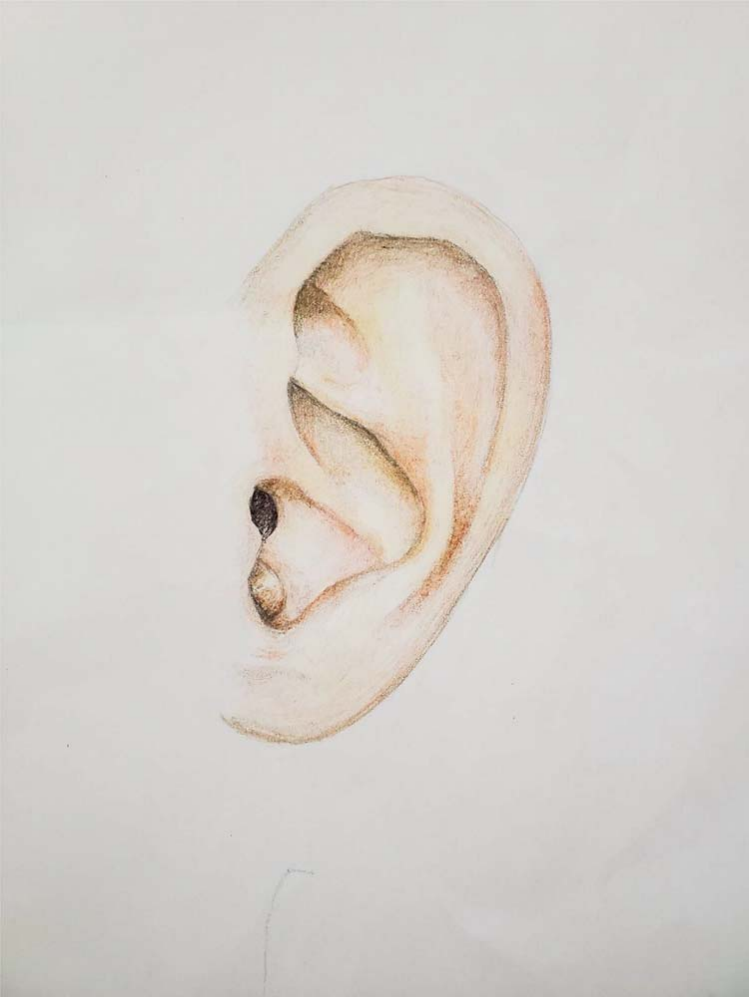
Medical illustration representing the location and macroscopic appearance of the lesion in the left auricle of the 51-year-old patient. Figure courtesy of M. Louka.

A 51-year-old male with unremarkable medical history presented to the Otolaryngology Clinic referred by his primary care physician with a small nodule on the left external auditory canal. The lesion was present for ~1 year, and the patient did not recall any history of local trauma or infections. He did not have hearing concerns. Microscopic otoscopy was performed in the office. The lesion appeared as a small, well-circumscribed nodule measuring ~5 mm in maximum diameter. It was skin-covered, dome shaped and had regular borders (Fig. 1). It felt rubbery on palpation with a cotton swab applicator. It was extending in the lateral and inferior aspect of the external auditory canal without completely occluding the introitus. The tympanic membrane was easily visualized and the examination was normal. The remaining head and neck examination and microscopic otoscopy of the contralateral ear were unremarkable. After informed consent was obtained, we proceeded to the operating room to excise the lesion under local anesthesia.

**Figure 2:**
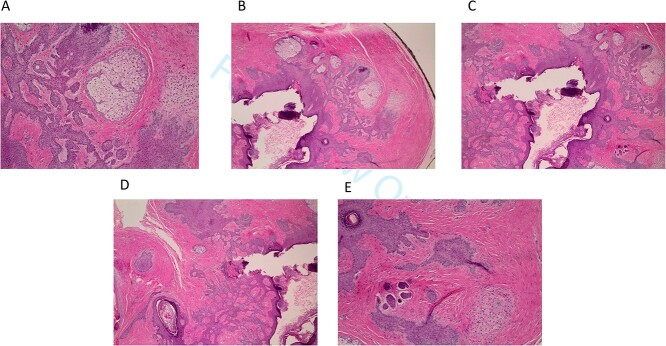
(**A–E**) Hematoxylin and eosin staining of excisional biopsy revealing trichofolliculoma pathologic characteristics at 200× magnification.

The procedure was performed with periauricular nerve block with lidocaine 1%/1:100 000 parts epinephrine under the surgical microscope. The lesion was removed in its entirety with the use of microsurgical instruments and a ‘beaver’ blade scalpel. It was found closely adherent to the cartilage of the external auditory canal. The incision on the ear canal skin was approximated with one single 5–0 plain gut absorbable suture. Histopathology revealed the diagnosis of TF following hematoxylin and eosin (H&E) staining (Fig. 2).

## DISCUSSION

TF is considered a rare, adnexal hamartomatous tumor originating from the hair follicles. It typically presents as a dome shaped, skin-colored nodule with a central pore showing protrusion of immature hair in the head and neck region in adults. The etiology of TF is unknown, although previous local trauma is considered to have a role [6]. Histologically, TF has been characterized by proliferated hair follicle stem cells, which are positive for cytokeratin 15 (CK15), and differentiate toward the outer root sheath while attempting to make hair [7].

The histopathological differential diagnosis of TFs includes hair follicle nevus, trichoepithelioma and accessory tragus [8]. These entities share a common histological background, with various amounts of mature hair follicles, and connective tissue framework in the subcutaneous fat. Central cysts associated with radiating hair follicles are more supportive of the diagnosis of TF [9].

TF is typically asymptomatic but surgical excision is indicated for biopsy purposes. Similar to previous cases, we conducted surgical excisional biopsy for this tumor. Despite its benign nature, there has been a report in the literature for a facial TF, with perineural invasion which may be indicative of more aggressive behavior [10]. In these patients, long-term follow-up is suggested in order to manage potential recurrence of the tumor. In our patient, the lesion had a slow gradual increase in size and was starting to occlude the external auditory canal, although no hearing loss was appreciated on first visit. In other cases, disfigurement of the auricle and esthetic concerns have been reported [4].

Although rare in the external auditory canal, TF should be included in the differential diagnosis of external auditory canal lesions. Its diagnosis is histopathological after excisional biopsy. Long-term follow-up will be required to rule out local recurrence.
